# Milk consumption and multiple health outcomes: umbrella review of systematic reviews and meta-analyses in humans

**DOI:** 10.1186/s12986-020-00527-y

**Published:** 2021-01-07

**Authors:** Xingxia Zhang, Xinrong Chen, Yujie Xu, Jie Yang, Liang Du, Ka Li, Yong Zhou

**Affiliations:** 1 West China School of Nursing/West China Hospital, Sichuan University, 37 Guo Xue Rd, Chengdu, 610041 China; 2 Department of Gastrointestinal Surgery, West China Hospital, Sichuan University, 37 Guo Xue Rd, Chengdu, 610041 Sichuan Province China; 3 Department of Public Health, School of Public Health, Sichuan University, Chengdu, 610041 China; 4 Chinese Evidence-Based Medicine/Cochrane Center, Chengdu, 610041 China

**Keywords:** Milk, Health, Umbrella review, Meta-analysis, Systematic review

## Abstract

In order to recapitulate the best available evidence of milk consumption and multiple health-related outcomes, we performed an umbrella review of meta-analyses and systematic reviews in humans. Totally, 41 meta-analyses with 45 unique health outcomes were included. Milk consumption was more often related to benefits than harm to a sequence of health-related outcomes. Dose–response analyses indicated that an increment of 200 ml (approximately 1 cup) milk intake per day was associated with a lower risk of cardiovascular disease, stroke, hypertension, colorectal cancer, metabolic syndrome, obesity and osteoporosis. Beneficial associations were also found for type 2 diabetes mellitus and Alzheimer's disease. Conversely, milk intake might be associated with higher risk of prostate cancer, Parkinson’s disease, acne and Fe-deficiency anaemia in infancy. Potential allergy or lactose intolerance need for caution. Milk consumption does more good than harm for human health in this umbrella review. Our results support milk consumption as part of a healthy diet. More well-designed randomized controlled trials are warranted.

## Introduction

Milk (*Lac*), which was used by human in the early of the seventh millennium BC [1, 2], is a nutritious, white liquid food secreted by the mammary glands of mammals. Cows' milk consumption varies around the world, with an average of 10–212 kg per person per year [3]. Milk contains 18 of 22 essential nutrients [4], including a various of bioactive peptides and fatty acids such as caseins, whey proteins, milk polar lipids (MPL), α-linolenic acid (ALA), conjugated linoleic acids (CLA), palmitic acid (16:0), lactose and other minor constituents (ie, calcium, phosphorous, magnesium, and vitamin D) which have an important impact on human metabolism and health [5, 6]. Evidence showed that milk has a wide range of physiological functionalities including anti-carcinogenic [7], anti-inflammatory [8], anti-oxidative [9], anti-adipogenic [10], anti-hypertensive [11], anti-hyperglycemia [12], and anti-osteoporosis [13]. Milk has been not only the primary source of nutrition for any newborn in mammalian species, but also an excellent source of the nutrients for children's growth and most adults, which has been recommended by the great amount of dietary guidelines all over the world [14, 15]. The American Heart Association/American College of Cardiology guidelines put forward that adults should intake three servings of dairy daily [16]. And the current Dietary Guidelines for Americans 2015–2020 for adults recommend the equivalent of three cups a day of fat-free milk [17].

The association of milk consumption and a sequence of health outcomes has been examined widely. However, the conclusions were inconsistent among different studies in humans [18–20]. In view of the importance of milk in our diet, it is crucial to consistently assess the totality of large amounts of data on the effects of milk intake on all health-related outcomes. Umbrella reviews could provide the highest quality of evidence, if performed and interpreted properly [21]. Thus, we conducted an umbrella review by integrating evidence from multiple meta-analyses to roughly generalize the advantages and disadvantages of milk consumption [22]. This way can help to determine the extent and magnitude of the connection of milk intake and different health outcomes, and more importantly, to evaluate the results of existing evidence for any risks that associated with increased milk consumption before an interventional trial was performed. And the results can provide the evidence which can be used to develop or renew dietary guidelines for decision makers.

## Methods

### Umbrella review methods

An umbrella review is the summary of existing systematic reviews and/or meta-analyses, which can present important information that can be used by decision makers in health care to systematically understand a topic area [23–25].

### Literature research

We search PubMed, Embase and Web of Science from the beginning to April 16, 2019 to identify the systematic reviews with meta-analyses of observational or interventional study that researched the connection of milk intake and multiple health-related outcomes. The following research strategy was used to conduct the literature retrieve: (milk OR dairy) AND (systematic review* OR meta-analys*), using truncated terminology for all areas. The reference lists of eligible papers and relevant clinical guidelines were also searched. Disagreements were resolved through consensus or discussion with the third researcher.

### Eligibility criteria

The inclusion criteria were as follows: (1) the article was a meta-analysis with/without systematic review of interventional and/or observational studies; (2) evaluated the association of milk consumption and health outcomes; (3) reported effect sizes: odds ratio (OR), relative risk (RR) or hazard ratio (HR) for qualitative outcomes and mean difference (MD) or standardized mean differences (SMD) for quantitative outcomes; (4) published in English. If there were more than one similar article, only the newest and larger one was included. The exclusion criteria were: (1) systematic reviews without meta-analyses; (2) data from animal or in vitro; (3) on dairy products.

### Data extraction

The processes of data extraction were performed by two authors independently. For individual eligible meta-analysis, the following information were extracted: first author, year, publication of journal, outcomes of interest, numbers of study and the type of milk. Then we extracted the amount of studies (which mean the number of study in the single meta-analysis included in our review), study designs (case–control, cohort, or randomized controlled trial [RCT]), and the number of cases and control/total participants. In addition, we abstracted data including metric (OR, RR, HR, MD, SMD), the summary estimates and related 95% confidence intervals (CI), heterogeneity (*I*^2^), fixed or random effect model was used in particular meta-analysis, and publication bias was recorded as well. If there were more than one outcome was reported in one article, we extracted each outcome respectively. If any discrepancies that were unable to be solved by consensus would be resolved by a third author, who made the final decision.

### Assessment of methodological quality and quality of evidence of included studies

The revised AMSTAR/AMSTAR 2 was used to assess the methodological quality of each involved meta-analysis, which was a trustworthy and well-founded measurement tool to estimate the levels of systematic reviews and meta-analysis for randomized and non-randomized studies [26]. The AMSTAR 2 was composed of 16 items including 7 critical domains and grades the overall confidence of each review as “high”, “moderate”, “low” and “critically low” based on detailed and specific explanations of bias. We used the GRADE system to assess the quality of data for included articles [27], which assorted the quality of data into four grade that “high”, “moderate”, “low”, and “very low”. Based on RCTs or observational studies, the grade of evidence can be decreased or increased according to the risk of bias, imprecision, inconsistency, indirectness, and magnitude of effect [28].

### Method of analysis

We extracted summary estimates and 95% CI of each related outcome, which was calculated by both fixed and inverse variance random effects methods. We extracted the *I*^2^ metric and Egger’s test to measure the heterogeneity and publication bias if they were available. And if the number of studies included in the meta-analyses was more than ten, we would calculate the publication bias through Egger`s regression test with the detailed original data were obtainable. A *P* < 0.1 for Egger`s regression test was regarded as the statistically significant publication bias. If the total estimate effects were not reported, we chose the outcomes derived from cohort rather than case–control or cross-sectional studies due to the quality of study. In dose–response analysis, the category of one serving or one glass of milk was equal to 244 g [29]. We did not reanalyze the other data or primary studied included in the meta-analysis.

## Results

### Characteristics of meta-analyses

**Fig. 1:**
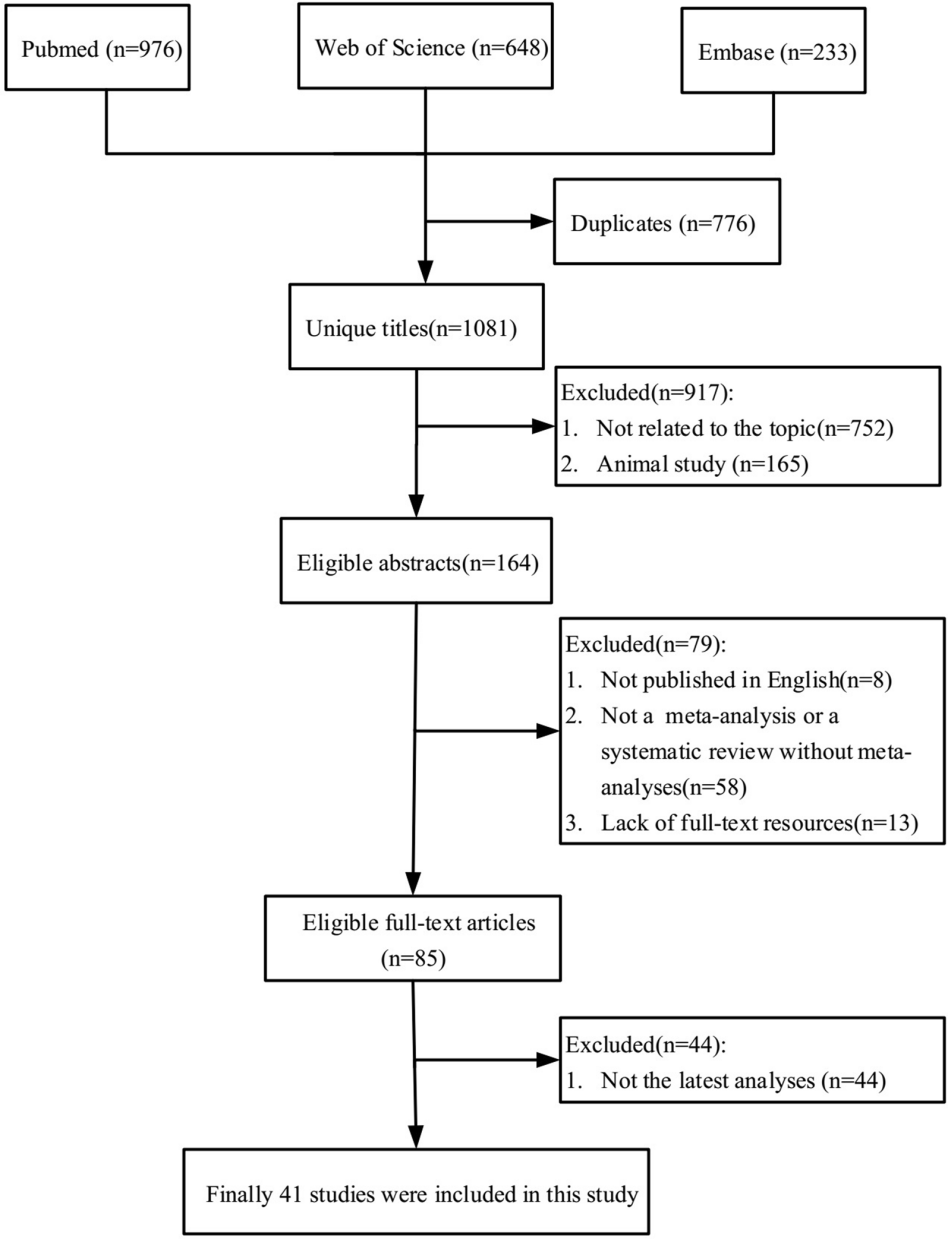
Flowchart of the selection process

**Fig. 2:**
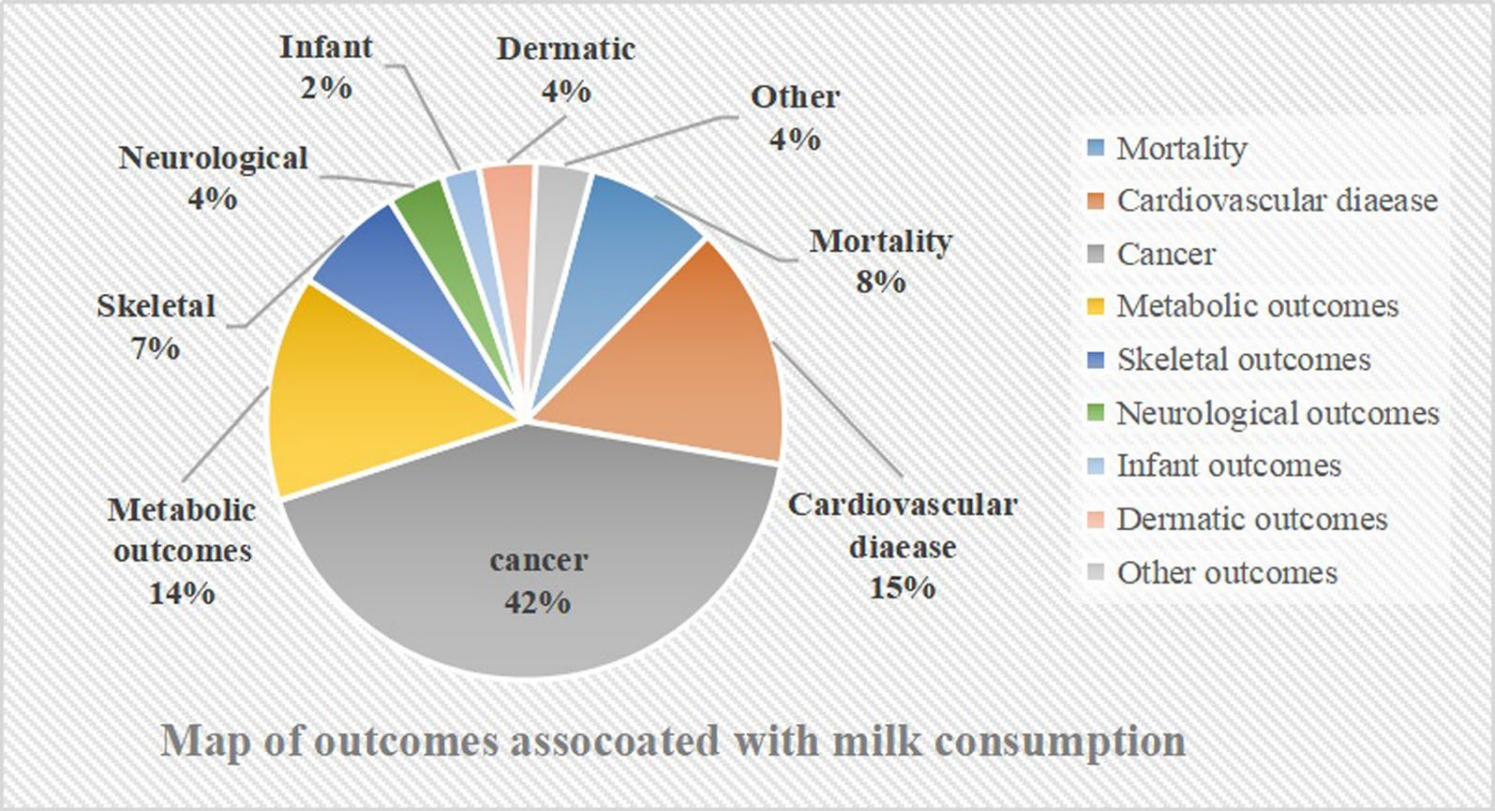
Map of outcomes associated with milk consumption

Figure 1 showed the processes of systematic search and results of eligible studies. Totally, 1857 articles were retrieved and 85 meta-analyses were eligible. Finally, forty-one most recent meta-analyses with 45 unique outcomes were included in our umbrella review (Fig. 2). The number of meta-analysis for single outcome ranged from one to seven and with a median number of two. The associations between milk intake and cancer outcomes were presented in Table 1. The relation of milk intake to mortality and cardiovascular disease (CVD) outcomes were shown in Table 2. And other outcomes related to milk consumption were shown in Table 3. The results of AMSTAR 2 and GRADE were shown in Table 4. Full versions of summary estimates which investigated the association between milk intake and all health-related outcomes were available in Additional file 1: Table S1.

**Table 1:** Associations between milk consumption and cancer outcomes

Outcomes	First author	Year	Types of milk	No. of studies in MA	Type of studies in MA	No. of cases/total	Effects mode	Metric of MA	Effect size	95% CI	*I*^2^%	Publication bias
**Significant associations**												
***Most beneficial***												
Colon cancer	Barrubes	2019	Low-fat milk	2	Cohort	3339/15,441	Fixed	RR	0.73	0.61–0.87	0	NA
Distal colon cancer	Barrubes	2019	Milk	3	Cohort	40,651/15,657	Fixed	RR	0.75	0.63–0.90	25	NA
CRC	Barrubes	2019	Low-fat milk	2	Cohort	3507/484,338	Fixed	RR	0.76	0.66–0.88	42	NA
Colon cancer	Barrubes	2019	Milk	8	Cohort	3339/15,441	Random	RR	0.79	0.72–0.87	0	NA
Proximal colon cancer	Barrubes	2019	Milk	3	Cohort	40,651/15,657	Fixed	RR	0.81	0.68–0.96	0	NA
CRC	Barrubes	2019	Milk	9	Cohort	9118/1,003,303	Random	RR	0.82	0.76–0.88	2	NA
Rectal cancer	Barrubes	2019	Milk	5	Cohort	NA	Random	RR	0.84	0.73–0.97	0	NA
Colon cancer	Barrubes	2019	Milk	3	Cohort	3339/15,441	Fixed	RR^a^	0.88	0.84–0.93	0	NA
Bladder Cancer	Bermejo	2019	Milk	14	Cohort/case control	NA/438,319	Random	RR	0.89	0.81–0.98	66.4	0.269
CRC	Barrubes	2019	Milk	9	Cohort	9118/1,003,303	Random	RR^b^	0.90	0.86–0.93	0	NA
Rectal cancer	Barrubes	2019	Milk	3	Cohort	NA	Fixed	RR^c^	0.91	0.84–0.97	25	NA
Prostate cancer	Aune	2015	Milk	8	Cohort	19,664/448,719	Random	RR	0.92	0.85–0.99	0	No
Breast cancer	Wu	2016	Skim milk	8	Cohort	16,664/586,726	Random	RR	0.93	0.85–1.00	40.1	0.616
Breast cancer	Wu	2016	Skim milk	5	Cohort	NA	Random	RR	0.96	0.92–1.00	11.9	0.498
***Most harmful***												
DLBCL	Wang	2016	Milk	3	Case–control	352/NA	Random	RR	1.49	1.08–2.06	8.9	NA
Gastric cancr	Wang	2018	Milk	21	Cohort/case control	NA	Random	RR	1.44	1.15–1.81	82.7	NA
NHL	Wang	2016	Milk	14	Cohort/case control	7109/NA	Random	RR	1.41	1.08–1.84	88.6	no
Ovarian cancer	Liu	2015	Milk	11	Cohort	NA	Random	OR	1.23	1.03–1.46	> 50	0.957
Bladder Cancer	Bermejo	2019	Milk	3	Cohort/case control	NA/3933	Random	RR	1.21	1.04–1.38	86.1	NA
Prostate cancer	Aune	2015	Low-fat milk	6	Cohort	19,430/432,943	Random	RR^d^	1.14	1.05–1.25	51	NA
NHL	Wang	2016	Milk	9	Cohort/case control	3739/NA	Random	RR^e^	1.13	1.00–1.28	NA	NA
NHL	Wang	2016	Milk	9	Cohort/case control	3739/NA	Random	RR^f^	1.12	1.00–1.26	NA	NA
Prostate cancer	Aune	2015	Milk	14	Cohort	11,392/566,146	Random	RR	1.11	1.03–1.21	21	no
Prostate cancer	Aune	2015	Low-fat milk	5	Cohort	NA/374,664	Random	RR^g^	1.06	1.01–1.11	67	NA
Prostate cancer	Aune	2015	Milk	13	Cohort	NA/559,383	Random	RR^g^	1.03	1.00–1.06	9	NA
**Non-significant associations**												
Distal colon cancer	Barrubes	2019	Milk	2	Cohort	40,651/15,657	Fixed	RR	0.78	0.60–1.01	0	NA
Breast cancer	Chen	2019	Low-fat milk	3	Case–control	NA	Random	OR	0.85	0.70–1.04	< 50	0.583
Colon cancer	Barrubes	2019	Milk	2	Cohort	3339/15,441	Fixed	RR	0.87	0.72–1.05	0	NA
Pancreatic cancer	Genkinger	2014	Low-fat milk	14	Cohort	307/NA	Random	HR	0.87	0.75–1.01	5	NA
Breast cancer	Wu	2016	Milk	18	Cohort	19,747/775,778	Random	RR	0.92	0.84–1.02	53.5	0.292
Pancreatic cancer	Genkinger	2014	Milk	14	Cohort	373/NA	Random	HR	0.92	0.77–1.10	0	NA
Ovarian cancer	Liu	2015	Low-fat/skim milk	13	Cohort	NA	Random	OR	0.93	0.79–1.09	< 50	0.370
ESCC	Li	2016	Milk	11	Case–control	2311/NA	Random	RR	0.93	0.74–1.16	52.9	0.960
Rectal cancer	Barrubes	2019	Milk	2	Cohort	NA	Fixed	RR	0.94	0.76–1.16	0	NA
Breast cancer	Chen	2019	Milk	8	Case–control	NA	Random	OR	0.95	0.80–1.13	< 50	0.272
CRC	Barrubes	2019	Milk	3	Cohort	5198/545,046	Fixed	RR	0.97	0.86–1.09	40	NA
Breast cancer	Wu	2016	Milk	11	Cohort	NA	Random	RR^g^	0.97	0.93–1.01	36.4	0.355
ESCC	Li	2016	Milk	6	Case–control	NA	Random	RR^b^	0.97	0.70–1.35	58.9	NA
Lung cancer	Yang	2016	Low-fat milk	3	Cohort/case control	NA	Random	RR	0.98	0.69–1.41	0	0.120
Prostate cancer	Aune	2015	Milk	6	Cohort	NA/388,664	Random	RR^g^	0.98	0.95–1.01	0	NA
NHL	Sergentanis	2019	Milk	4	Cohort	1517/NA	Random	RR	0.99	0.85–1.15	0	0.461
Breast cancer	Wu	2016	Milk	9	Cohort	13,781/554,775	Random	RR	0.99	0.87–1.12	37.4	0.723
Endometrial cancer	Li	2017	Milk	6	Cohort/case control	3538/331,168	Random	OR	0.99	0.89–1.10	0	NA
FL	Wang	2016	Milk	3	Case–control	390/NA	Random	RR	0.99	0.47–2.07	89.8	NA
Pancreatic cancer	Genkinger	2014	Milk	14	Cohort	145/NA	Random	HR	1.01	0.83–1.22	0	NA
Breast cancer	Wu	2016	Milk	5	Cohort	NA	Random	RR^g^	1.02	0.92–1.13	32.8	0.660
NHL	Wang	2016	Milk	9	Cohort/case control	3739/NA	Random	RR^h^	1.04	0.97–1.12	NA	NA
SLL/CLL	Wang	2016	Milk	3	Case–control	477/NA	Random	RR	1.04	0.69–1.55	44.1	NA
NHL	Wang	2016	Milk	9	Cohort/case control	3739/NA	Random	RR^i^	1.07	0.96–1.19	NA	NA
Lung cancer	Yang	2016	Milk	22	Cohort/case control	NA	Random	RR	1.08	0.80–1.46	90.5	0.300
NHL	Wang	2016	Milk	9	Cohort/case control	3739/NA	Random	RR^j^	1.11	0.99–1.24	NA	NA
HCC	Yang	2017	Milk	7	Cohort/case control	NA	Random	RR	1.13	0.67–1.88	78	> 0.1
Proximal colon cancer	Barrubes	2019	Milk	2	Cohort	40,651/15,657	Fixed	RR	1.20	0.96–1.49	83	NA

*MA* meta-analysis, *CI* confidence interval, *RR* risk ratio, *HR* hazard ratio, *OR *odds ratio, *NHL* non-Hodgkin’s lymphoma, *HCC* hepatocellular carcinoma, *CRC* colorectal cancer, *DLBCL* diffuse large B-cell lymphoma, *FL* follicular lymphoma, *SLL/CLL* small lymphocytic lymphoma/chronic lymphocytic leukemia, *ESCC* esophageal squamous cell carcinoma, *NA* not available

^a^488 g/day; ^b^244 g/day; ^c^732 g/day; ^d^highest verse lowest; ^e^200 g/day; ^f^440 g/day; ^g^490 g/day; ^h^120 g/day; ^i^210 g/day; ^j^370 g/day

**Table 2:** Association between milk consumption and mortality and cardiovascular disease

Outcomes	First author	Year	Types	No. of studies in MA	Type of studies in MA	No. of cases/total	Effects mode	Metric of MA	Effect size	95% CI	*I*^2^%	Publication bias
**Mortality**												
*Significant associations*												
CHD mortality	Mazidi	2018	Milk	3	Cohort	18,927/105,528	Random	RR	1.04	1.02–1.06	10.4	NA
PC mortality	Lu	2016	Milk	NA	Cohort	NA	Random	RR	1.50	1.03–2.17	NA	NA
PC mortality	Lu	2016	Milk	NA	Cohort	NA	Random	RR^a^	1.43	1.13–1.81	NA	NA
*Non-significant associations*												
CVD mortality	O'Sullivan	2013	Milk	7	Cohort	17,455/338,421	Random	RR	0.96	0.81–1.13	22.8	NA
All-cancer mortality	Lu	2016	Milk	NA	Cohort	NA	Random	RR	0.97	0.92–1.03	8.4	0.95
All-cause mortality	Mazidi	2018	Milk	3	Cohort	23,324/63,390	Random	RR	0.99	0.98–1.00	8.3	NA
PC mortality	Lu	2016	Skim/low-fat milk	NA	Cohort	NA	Random	RR	1.00	0.75–1.33	NA	NA
Total mortality	Mullie	2016	Milk	11	Cohort	63,545/281,788	Random	RR^a^	1.01	0.96–1.06	94	0.64
**Cardiovascular outcomes**												
*Significant associations*												
Stroke	de Goede	2016	Milk	14	Cohort	25,269/603,920	Random	RR^b^	0.93	0.88–0.98	86	0.06
CVD	Soedamah-M	2011	Milk	4	Cohort	2283/13,518	Random	RR^b^	0.94	0.89–0.99	0	NA
Hypertension	Soedamah-M	2012	Milk	7	Cohort	14,398/47,647	Random	RR^b^	0.96	0.94–0.98	NA	NA
Stroke	de Goede	2016	High-fat milk	4	Cohort	5942/159,547	Random	RR^b^	1.04	1.02–1.06	0	NA
*Non-significant associations*												
Arterial Stiffness	Diez-F	2019	Milk	4	Cross-sectional	NA/15,553	Random	NA	0.02	-0.01–0.05	0	NA
Stroke	Gholami	2017	Milk	10	Cohort	22,946/440,397	Random	RR	0.91	0.81–1.01	71.4	0.45
Stroke	de Goede	2016	Low-fat milk	4	Cohort	5942/159,547	Random	RR^b^	0.96	0.90–1.03	68.2	NA
CVD	Guo	2017	Milk	9	Cohort/case control	21,580/249,779	Random	RR	1.01	0.93–1.10	92.4	No
CHD	Gholami	2017	Milk	9	Cohort	4866/212,767	Random	RR	1.05	0.96–1.15	0	0.6

*MA* meta-analysis, *No*. number, *CI* confidence interval, *RR* risk ratio, *CHD* coronary heart disease, *PC* prostate cancer, *CVD* cardiovascular disease, *NA* not available

^a^244 g/day; ^b^200 g/day

**Table 3:** Association between milk consumption and metabolic, skeletal, cognitive, infant and other outcomes

Outcomes	First author	Year	Types	No. of studies in MA	Type of studies in MA	No. of cases/total	Effects mode	Metric of MA	Effect size	95% CI	*I*^2^%	Publication bias
***Significant associations***												
*Most beneficial*												
Osteoporosis	Malmir	2019	Milk	6	Cohort	NA	Random	RR^a^	0.61	0.50–0.75	NA	no
Alzheimer's disease	Wu	2016	Milk	2	Cohort	417/NA	Random	OR	0.63	0.44–0.90	0.0	NA
Cognitive Disorders	Wu	2016	Milk	5	Cohort/cross-sectional	1273/NA	Random	OR	0.72	0.56–0.93	64.0	NA
Metabolic syndrome	Mena	2019	Milk	5	Cohort	4065/15,657	Random	RR	0.79	0.64–0.97	66.0	NA
Obesity	Wang	2016	Milk	16	Cohort/cross-sectional	NA	Random	OR	0.81	0.75–0.88	62.1	0.109
Obesity	Wang	2016	Milk	4	Cohort/cross-sectional	NA	Random	OR^a^	0.84	0.77–0.92	NA	NA
Metabolic syndrome	Lee	2018	Milk	9	Cohort/case control	7002/29,077	Random	RR^a^	0.87	0.79–0.95	44.7	0.2
T2DM	Tian	2017	Milk	7	Cohort	NA	Random	RR	0.87	0.78–0.96	52.2	0.67
Abdominal obesity	Lee	2018	Milk	7	Cohort/case control	NA	Random	RR^a^	0.88	0.79–0.97	56.8	NA
HTG	Lee	2018	Milk	4	Cohort/case control	NA	Random	RR^b^	0.90	0.81–0.98	0.0	NA
*Most harmful*												
FDA	Griebler	2016	Milk	4	RCT/cohort	NA/1683	Random	RR	3.67	2.73–5.19	0.0	NA
Acne	Aghasi	2019	Milk	8	Cohort/case control/cross-sectional	3102/19,376	Fixed	OR	1.48	1.31–1.66	23.6	0.17
PD	Jiang	2014	Milk	5	Cohort	873/304,193	Random	RR^b^	1.45	1.23–1.73	16.1	0.62
Acne	Juhl	2018	Milk	3	Cohort	7856/53,214	Random	OR^c^	1.41	1.05–1.90	NA	NA
PD	Jiang	2014	Milk	4	Cohort	785/278,786	Random	RR^a^	1.17	1.06–1.30	NA	NA
Hip fracture	Malmir	2019	Milk	8	Cohort	NA	Random	RR^a^	1.09	1.07–1.11	NA	0.015
***Non-significant associations***												
Dental erosion	Li	2012	Milk	4	Cohort	NA/3387	Random	OR	0.67	0.11–4.01	NA	NA
Dementia	Wu	2016	Milk	3	Cohort/cross-sectional	552/NA	Random	OR	0.70	0.48–1.02	18.0	NA
Osteoporosis	Malmir	2019	Milk	6	Cohort/case control/cross-sectional	NA	Random	RR	0.79	0.57–1.08	63.3	no
Vertebral fracture	Matia	2019	Milk	3	Cohort	NA/15,295	Random	HR	0.81	0.66–1.00	0.0	0.068
HDL-C	Lee	2018	Milk	4	Cohort/case control	NA	Random	RR^b^	0.89	0.75–1.04	72.8	NA
Endometriosis	Hoorsan	2017	Milk	2	Cohort/case control	1862/69,702	Random	OR	0.90	0.65–1.23	81.2	0.32
Hip fracture	Malmir	2019	Milk	10	Cohort	NA	Random	RR	0.93	0.75–1.15	86.7	0.015
T2DM	Gijsbers	2016	Milk	11	Cohort	17,241/145,472	Random	RR^a^	0.97	0.93–1.02	57.0	0.07
T2DM	Gijsbers	2016	High-fat milk	9	Cohort	267,588/336,061	Random	RR^a^	0.99	0.88–1.11	84.0	0.78
T2DM	Gijsbers	2016	Low-fat milk	7	Cohort	200,981/267,588	Random	RR^a^	1.01	0.97–1.05	72.0	NA
Cognitive function	Lee	2018	Milk	3	Cohort	714/5460	Random	RR	1.21	0.81–1.82	64.1	NA

*MA* meta-analysis, *CI* confidence interval, *RR* risk ratio, *OR* odds ratio, *HR* hazard ratio, *MD* mean difference, *PD* Parkinson’s disease, *FDA* Fe-deficiency anaemia, *T2DM* type 2 diabetes mellitus, *HTG* hypertriacylglycerolaemia, *HDL-C* high-density lipoprotein cholesterol, *NA* not available

^a^200 g/day; ^b^highest versus lowest; ^c^≤ 1 glass/week versus 1 glass/day

**Table 4:** Assessments of AMSTAR 2 scores and GRADE classification

Outcomes	First author	Year	Types	AMSTAR 2	GRADE
**Mortality**					
All-cause mortality	Mazidi	2018	Milk	Moderate	Low
CHD mortality	Mazidi	2018	Milk	Moderate	Low
All cancer mortality	Lu	2016	Milk	High	Low
Prostate cancer mortality	Lu	2016	Milk	High	Very low
Prostate cancer mortality	Lu	2016	Skim/low-fat milk	High	Very low
**Cancer**					
CRC	Barrubes	2019	Milk	Low	Moderate
CRC	Barrubes	2019	Low-fat milk	Low	Low
Prostate cancer	Aune	2015	Milk	Low	Moderate
DLBCL	Wang	2016	Milk	Critically low	Low
Gastric cancr	Wang	2018	Milk	Critically low	Moderate
Bladder Cancer	Bermejo	2019	Milk	Moderate	Moderate
Bladder Cancer	Bermejo	2019	Whole milk	Moderate	Low
Breast cancer	Chen	2019	Low-fat milk	Critically low	Very low
Breast cancer	Chen	2019	Milk	Critically low	Low
Breast cancer	Wu	2016	Milk	Moderate	Moderate
Breast cancer	Wu	2016	Skim milk	Moderate	Moderate
Endometrial cancer	Li	2017	Milk	Moderate	Moderate
ESCC	Li	2016	Milk	High	Moderate
FL	Wang	2016	Milk	Critically low	Very low
HCC	Yang	2017	Milk	Low	Moderate
Lung cancer	Yang	2016	Milk	Moderate	Moderate
Lung cancer	Yang	2016	Low-fat milk	Moderate	Low
NHL	Sergentanis	2019	Milk	Low	Low
Ovarian cancer	Liu	2015	Low-fat/skim milk	Critically low	Low
Ovarian cancer	Liu	2015	Milk	Critically low	Moderate
Pancreatic cancer	Genkinger	2014	Milk	Critically low	Moderate
Pancreatic cancer	Genkinger	2014	Whole milk	Critically low	Moderate
Pancreatic cancer	Genkinger	2014	Low-fat milk	Critically low	Moderate
SLL/CLL	Wang	2016	Milk	Critically low	Very low
**Cardiovascular outcomes**					
CVD	Guo	2017	Milk	High	Low
CVD	Soedamah-Muthu	2011	Milk	Low	Low
CHD	Gholami	2017	Milk	Moderate	Moderate
Arterial Stiffness	Diez-Fernandez	2019	Milk	High	Moderate
Hypertension	Soedamah-Muthu	2012	Milk	Low	Moderate
Stroke	de Goede	2016	Milk	Moderate	Moderate
Stroke	de Goede	2016	High-fat milk	Moderate	Low
Stroke	de Goede	2016	Low-fat milk	Moderate	Low
Stroke	Gholami	2017	Milk	Moderate	Moderate
**Metabolic outcomes**					
Abdominal obesity	Lee	2018	Milk	Moderate	Moderate
T2DM	Gijsbers	2016	Milk	Moderate	Low
T2DM	Gijsbers	2016	Low-fat milk	Moderate	Low
T2DM	Gijsbers	2016	High-fat milk	Moderate	Low
Hypertriacylglycerolaemia	Lee	2018	Milk	Moderate	Low
Metabolic Syndrome	Mena	2019	Milk	Critically low	Moderate
Metabolic Syndrome	Lee	2018	Milk	Moderate	Moderate
Obesity	Wang	2016	Milk	Low	Moderate
T2DM	Tian	2017	Whole milk	Low	Low
**Skeletal outcomes**					
Hip fracture	Malmir	2019	Milk	Low	Low
Osteoporosis	Malmir	2019	Milk	Low	Moderate
Vertebral fracture	Matia	2019	Milk	Low	Very low
**Neurological outcomes**					
Alzheimer's disease	Wu	2016	Milk	Moderate	Very low
Cognitive Disorders	Wu	2016	Milk	Moderate	Low
Cognitive function	Lee	2018	Milk	Moderate	Very low
Parkinson’s disease	Jiang	2014	Milk	Low	Low
Dementia	Wu	2016	Milk	Moderate	Very low
**Infant outcomes**					
FDA	Griebler	2016	Milk	Low	Low
T1DM	Griebler	2016	Milk	Low	Low
**Other outcomes**					
Acne	Aghasi	2019	Milk	Low	Moderate
Dental erosion	Li	2012	Milk	Critically low	Very low
Endometriosis	Hoorsan	2017	Milk	Low	Very low

*AMSTAR* a measurement tool to assess systematic reviews, *GRADE* Grading of Recommendations Assessment, Development, and Evaluation, *CVD* cardiovascular disease, *CHD* coronary heart disease, *CRC* colorectal cancer, *DLBCL* diffuse large B-cell lymphoma, *ESCC* esophageal squamous cell carcinoma, *FL* follicular lymphoma, *HCC* hepatocellular carcinoma, *NHL* non-Hodgkin’s lymphoma, *SLL/CLL* small lymphocytic lymphoma/chronic lymphocytic leukemia, *T2DM* type 2 diabetes mellitus, *FDA* Fe-deficiency anaemia, *T1DM* type 1 diabetes mellitus

### Mortality

Milk consumption was not connected with total mortality [30], CVD mortality [31] or all-cancer mortality [32], while it was associated with a elevated risk of mortality from coronary heart disease (CHD) (1.04; 1.02–1.06) [30] and prostate cancer (1.50; 1.03–2.17) [32].

### Cardiovascular disease

Although high verse low milk consumption was not related to the risk of CVD, CHD and stroke [33, 34], dose–response analysis manifested a 7% lower risk of stroke (0.93; 0.88–0.98) [35], a 6% lower risk of CVD (0.94; 0.89–0.99) [36], and a 4% lower risk of hypertension (0.96; 0.94–0.98) [37] with increment of 200 ml milk consumption per day. However, high-fat milk intake was connected with a 4% higher risk of stroke (1.04; 1.02–1.06) [35].

### Cancer outcomes

High milk intake was consistently related to decreased risk of colorectal cancer (CRC) (0.82, 0.76–0.88) [38]. The meta-analysis with 1,003,303 subjects showed that the highest milk intake was connected with a lower risk of both colon and rectal cancer, especially in colon cancer (0.79; 0.72–0.87) [38]. However, the effects depend on the types of milk. Low-fat milk consumption was significantly related to decreased risk of CRC. Dose–response analysis showed that there was a significant linear association and per 1 serving increment of total milk was connected with a 10% lower risk of CRC [38].

Conversely, compared with low milk consumption, high consumption were related to increasing risk of prostate cancer (1.11; 1.03–1.21) [39], diffuse large B-cell lymphoma [40] and gastric cancer [41]. A 200 g/day milk consumption was connected with increasing risk of prostate cancer and the summary relative risk was 1.03 (95% CI 1.00–1.06; *P* = 0.04) [39].

The effects were inconsistent for bladder cancer [42], breast cancer [43], ovarian cancer [44] and non-Hodgkin’s lymphoma [40] because of the different type or dose of milk. No association was found between milk consumption and endometrial cancer [45], esophageal squamous cell carcinoma [46], hepatocellular carcinoma [47], lung cancer [48], follicular lymphoma [40], small lymphocytic lymphoma/chronic lymphocytic leukemia [40] and pancreatic cancer [49].

### Metabolic outcomes

Higher milk intake was contrarily related to the T2DM risk (0.87; 0.78–0.96) [50], metabolic syndrome (0.79; 0.64–0.97) [51] and obesity (0.81; 0.75–0.88) [52]. Dose–response analysis suggested that the 200 g/day increment of milk was related to a 13% lower risk of metabolic syndrome [53] and a 16% lower risk of obesity [52].

### Skeletal outcomes

Milk consumption was not related to the risk of hip fracture [54] while every additional 200 g/day milk consumption was connected with a 39% lower risk of osteoporosis (0.61; 0.50–0.75) [55].

### Neurological outcomes

High milk intake was connected with a decreased risk of Alzheimer's disease (AD) (0.63; 0.44–0.90) [56], but it was connected with the increased risk of Parkinson’s disease (PD) (1.45; 1.23–1.73) [57]. Linear dose–response relationship manifested that PD risk would be increased by 17% for every 200 g/day per day increase in milk consumption [57].

### Infant outcomes

High milk consumption was related to an elevated risk of developing Fe-deficiency anaemia (3.67; 2.73–5.19) [58] but not of type 1 diabetes mellitus [58] in infancy.

### Other outcomes

Milk intake was positively connected with the increased risk of acne (1.48; 1.31–1.66) [59] but not with endometriosis [60] or dental erosion [61].

### Side effects

The prevalence of cow's milk allergy was 0.6–3.0% by sensitization tests or challenge confirmed allergy [62, 63]. Immunotherapy is promising (in terms of acquiring desensitization) but data are insufficient to recommend use [63–65]. Lactose intolerance is a real and important clinical syndrome [66, 67], its prevalence is 0–17.9% [68]. However, most person with presumed lactose intolerance or malabsorption can tolerate 12–15 g of lactose (roughly 1 cup of milk) [67, 69].

### Heterogeneity of included studies

In the all included studies, about 37.8% studies had a lower heterogeneity with *I*^2^ < 25%; about 31.6% studies had a moderated heterogeneity, the *I*^2^ between 25 and 75%; and 14.3% studies had a high heterogeneity with *I*^2^ > 75%. However, there were 16.3% studies did not reported the heterogeneity and we cannot re-analysis because of the unavailable information.

### Publication bias of included studies

The funnel plots and Egger’s test were used in this umbrella. About 31.6% studies reported there were no publication biases while 5 report significant evidence for publication biases including stroke, hip fracture, vertebral fracture and diabetes [70]. The others meta-analysis did not reported the outcomes of publication bias owe to the insufficient number of studies. However, it was very possible that unreported publication bias existed in many of the included studies.

### AMSTAR 2 and GRADE classification of included studies

The results of AMSTAR 2 of the included studies were shown in Table 4. The studies were rated as four levels, and 11.1% were rated as “high”, about 30.6% were rated as “moderate”, about 38.9% were rated as “low” and 19.4% were classified into “critically low”. And the reason was that most of studies failed to report the funding sources of the studies included in the meta-analysis (item 10). The detailed results of each item of AMSTAR 2 for the included meta-analysis were available in Additional file 2: Table S2. As for the quality of outcomes, about 18.4% were graded as “very low”, forty percent were graded as “low” and 41.6% were graded as “moderate”. None one was stratified as “high” because the meta-analyses were derived from observational study and most of them came from subgroup with a limited sample size, risk of bias, inconsistency or imprecision. The detailed information about GRADE was shown in Table 4.

## Discussion

### Main findings and possible explanations

- We totally identified 41 meta-analyses with 45 unique outcomes in this umbrella review. According to the existing evidence, milk consumption was more often associated with benefits than harm to a sequence of health-related outcomes. Beneficial associations were found for CVD, stroke, hypertension, CRC, metabolic syndrome, obesity, osteoporosis, T2DM and AD. However, high intake of milk might slightly increase the risk of prostate cancer, PD, acne and Fe-deficiency anaemia in infancy. Side effects including allergy and lactose intolerance need for caution. Dairy products (such as cheese, butter and others) and milk form other species (human, formula milk and donkey, ovine and caprine) consumption was not included in this review because of the complex and different nutritional ingredients.

Milk intake was connected with a lower incidence of CVD in this umbrella review. In the early 1985, the CARDIA study of 4304 participants has indicated that intakes of milk was inversely associated with the elevated blood pressure (BP) over a 15-year follow-up period [71]. RCTs have shown that milk proteins can significantly reduce the systolic BP, diastolic BP, 24-h ambulatory BP, and other risk markers for CVD including total cholesterol (TC) and triacylglycerol [72, 73]. It has been considered that milk fats were important sources for saturated fatty acids (SFAs), which have been related to an elevated risk of CVD because of the high levels of low density lipoprotein cholesterol (LDL-C), therefore, low-fat or fat-free milk rather than regular-fat milk was recommended by some authorities and guidelines [16, 17, 74]. However, outcomes from short-term interventional studies about CVD bio-markers have demonstrated that whole-fat milk would increase LDL-C, while high density lipoprotein cholesterol (HDL-C) was increased as well, and therefore might not influence or even lower the ratio of TC: HDL-C [75]. And a randomized crossover study has found that the differences of whole milk and skimmed milk for TC, LDL-C and triacylglycerol were not significant [76]. In addition, an international collaboration proposed that 2018 World Health Organization draft guideline on dietary SFAs of reducing consumption total of SFAs would be overthrown because which failed to take into account considerable evidence [77]. The mechanisms may be depend on the various components of milk. (1) SFAs (such as C15 and C17) may have a protective effect on CVD in observational studies [78, 79]; (2) CLA and sphingolipids had potential cardio-protective effects [80]; (3) Milk proteins can be digested and generated the bioactive peptides, which were connected with a decreasing hypertension risk [81]; (4) Higher Calcium intake was associated with decreased concentrations of total-C and LDL-C [82], which may have a positive impact on blood lipids, because Ca intake was related to the excretion of fat in the faeces [82]; (5) Milk-derived tripeptides had BP-lowering effects [83]; (6) Notably, the emerging functional ingredient MPL, which are nature component of the milk fat globule membrane [10], can significantly reduce the lipid biomarkers of CVD, including TC/HDL-C and apolipoprotein (Apo)B/ApoA1 ratios by reducing intestinal cholesterol absorption [84]. All of the evidence showed that milk consumption would not rise up the risk of CVD, whereas it may show a protective effect in CVD, which can be included as part of healthy diet [85].

The meaningful finding of this umbrella review was that milk consumption decreased the risk of CRC. Previously in 1977, it has been proposed that higher intake of milk had a protective effect on colon cancer [86]. A recent cohort study included 77,712 Seventh-day Adventists over a mean follow-up 7.8 years has found that milk intake might decrease the risk of CRC [87]. The study of 477,122 participants over a mean follow-up 11 years also found that both whole-fat milk and skimmed milk intake were inversely connected with risk of CRC [84]. The Norwegian Women and Cancer Cohort Study of 81,675 participants indicated that milk consumption was weakly associated with a lower risk of colon cancer among women [88]. Furthermore, milk intake was connected with the mortality of patients with CRC. Yang et al*.* performed a prospective cohort study with 2284 participants who were diagnosed with invasive non-metastatic CRC proved that post-diagnosis milk consumption was inversely connected with a lower all-cause mortality [89]. Several possible biological mechanisms might underlie the associations: (1) Calcium, the main component of milk can unconcerned about bile acids and FFAs (predominately deoxycholic and lithocolic acids) and prevent or reduce their toxicity to the colonic epithelial cells [90]; (2) Vitamin D would protect against colon cancer, it has been found that higher serum 25-hydroxyvitamin D was related to a decreasing risk of colon cancer [91]; (3) The subtypes of dairy fat could inhibit colorectal carcinogenesis, such as: CLA can inhibit CRC cells growth in vitro [92], and the butyric acid can hamper proliferation and bring about differentiation of tumor cell lines in vitro [90]; (4) The bovine lactoferrin can inhibit CRC and significantly retarded adenomatous colorectal polyp growth [93]; (5) Low-fat milk consumption can reduce the risk of CRC by 60%, especially among individuals with high IGF-1/IGF-binding protein-3 [94]. The WCRF/AICR reported the conclusion of milk consumption probably protected against colorectal cancer [95].

High milk intake was related to an elevated risk of prostate cancer and prostate cancer mortality in our umbrella review. In the early 1984, the associations between prostate cancer and milk consumption have been found [96]. In the later, a prospective cohort study with 25,708 participants followed by 12.4 years found that skim milk consumption was associated with a significantly increased risk of prostate cancer compared with whole milk consumption [97]. The reason was that skim milk was significantly positively associated with BMI [97], and body mass would have an influence on serum androgen concentrations [96]. Recently, the similar results have been found in a multiethnic cohort study with 82,483 men [98]. They suggested that the associations of prostate cancer with milk consumption might vary because of fat content, particularly for the early formation of the cancer [98]. Most interesting, Torfadottir et al. found that high milk consumption in early life (aged 14–19 years) was related to a 3.2-flod risk of advanced prostate cancer after adjusting lifestyle and other factors [99]. In addition, milk consumption was associated with the recurrence and progression of prostate cancer as well. A prospective article with 1334 men confirmed that whole milk consumption more than four servings per week would increase the risk of recurrence by 85% for patients with non-metastatic cancer compared with less three servings a month [100]. Milk consumption after diagnosis was related to a worse progression, Downer et al. conducted a 20-year follow-up study with 525 men who were recently diagnosed with prostate cancer and found that high-fat milk consumption more than 3 servings daily was associated with higher risk of mortality from prostate cancer among agents with localized prostate cancer compared with the low volume consumers [101]. The following mechanisms have been proposed: (1) Milk consumption was associated with higher circulating IGF-1 levels may be in line with the risk of prostate cancer [102]. Each 200 g increment in milk per day was related to 10.0 ug/L higher IGF-1 [102]; (2) The casein would contribute to the proliferation of prostate cancer cells including PC3 and LNCaP [103]; (3) Milk would disrupt the p53 and DNA methyltransferase 1 and promote prostate cancer, which were the guardians of the genome [104]; (4) Calcium and phosphorous may decrease concentrations of 1,25(OH)2D, which can inhibit the carcinogenesis of prostate and contribute to apoptosis [101]. An overview [105] and the WCRF/AICR report [106] concluded that milk consumption probably increased prostate cancer risk, while the evidence was limited.

Beneficial associations were found between milk consumption and metabolic syndrome, T2DM, and obesity. The cohort studies with 7240 adults in Korean found that the people consumed more than seven servings per week had a half reduction of metabolic syndrome risk, and the individual components such as elevated blood pressure, hypertriacylglycerolaemia, abdominal obesity and hyperglycaemia were reduced as well compared with non-drinkers [107]. Another prospective cohort study with 63,257 Chinese people found that high milk consumption was significantly connected with a 12% decrease in the risk of T2DM [108]. And the effects were increased with the volume of milk consumption, A prospective cohort study (Shanghai Women’s Health Study), based on population with 64,191 women aged 40–70 years from 7 urban communities in Shanghai, found that the associations followed a dose-dependent relationship, the HR of T2DM was 0.61 for < 100 g/day, 0.56 for 100–200 g/day, and 0.46 for > 200 g/day milk consumption compared with non-consumers [109]. Besides, milk consumption was also inversely associated with obesity, each increment 100 ml/d was associated with 0.26 kg/m^2^ lower BMI [83]. A meta-analysis of 37 RCTs manifested that high dairy intake was associated with lower body weight and body fat while higher lean mass with energy restriction [110]. The main components of milk such as calcium and magnesium [109], Casein and whey protein [5], *trans*-11 vaccenic acid [111], linoleic acid [112], MPL [10], vitamin D [113], and its effect on enhancing satiety [114] may be responsible for the mechanism behind the beneficial associations.

The associations between milk consumption and neurological outcomes were mixed in this article. Milk consumption was beneficial to AD while being harmful for PD. Prospective cohort study (the Hisayama Study) with 1018 elderly Japanese over 17 years of follow-up has found that greater milk intake reduced the risk of dementia, especially AD with a linear relationship [115]. The possible mechanisms were proposed that milk and its components such as milk peptide [116], β-Casein [117], calcium and magnesium [115], would tribute to the low risk of AD by suppressing the expression of inflammatory cytokines and production of oxidative stress [118], inhibiting the aggregation and deposition of Aβ1-42 fibrils [117] and other mechanisms. However, several prospective cohort studies (such as the Nurses' Health Study, the Health Professionals Follow-up Study) have found that high milk consumption was associated with elevated risk of PD [119], and the risk of PD was 2.3-fold in the highest group (sixteen Ounces per day) compared with lowest group in the Hnolulu Heart Program [120]. But there were no clear explanations for the associations. Possible explanations included pesticides residues in milk such as organochlorine and tetrahydroisoquinoline [121], and milk protein casein may increase the risk of PD by reducing serum urate or uric acid concentrations [122]. Based on currently evidence, limiting the consumption of milk was not a reasonable strategy in the prevention of PD [123].

Milk intake might increase the risk and severity of acne in this review [59]. A Norwegian longitudinal study in 2489 adolescence found that high consumption of milk would increase the risk of acne in girls but not in boys [124]. The gender differences would be due to the different pattern of dairy intake, maturational stage and life styles [124]. Another recent meta-analysis of observational studies in individuals aged 7–30 years also demonstrated milk consumption was related to a higher risk of acne, not only for whole milk but also low-fat or skimmed milk, and the effects were significantly related to the frequency of milk consumption [125]. The possible explanation was that milk would increase the insulin and IGF-1 concentration [102] which would promote the phosphorylation of transcription factor Forkhead box protein O1, trigger the nutrient sensitive kinase, mammalian target of rapamycin complex 1, stimulate the sebaceous glands and result in occurrence of acne [126, 127]. However, the Mendelian randomization study with 20,416 Danish adults failed to observe the associations between milk consumption and acne [128]. Therefore, more RCTs are needed in the further research to clarify the causal association especially in adolescence.

Cow’s milk consumption was related to over three-fold risk of Fe-deficiency anaemia in infancy compared with those who consumed follow-on formula in our review. Summary analysis from of cohorts has revealed that the incidence of iron deficiency was highest in cow’s milk group compared with breast milk or follow-on formula [129]. A double-blind RCT showed that the prevalence of Fe-deficiency anaemia was 33% in cow’s milk group while 2% in iron supplemented group [130]. Several mechanisms have been identified: (1) The most important was the low iron content (0.5 mg/L) of cow’s milk [131]; (2) Milk consumption during infancy would result in occult intestinal blood loss [132]; (3) The components of milk including calcium and casein would inhibit the absorption of non-heme iron [131, 133]. Fe-fortified milk or follow-on formula would be efficacious ways to prevent the occurrence of Fe-deficiency anaemia [130].

Milk allergy has been described in modern literature by Hamburger in 1901 [134]. In the later, antigens in cow's milk were identified [135]. Recently, several approaches were found to prevent and treat milk allergy [136, 137]. The notion of lactose intolerance can date back to the mid-twentieth century when the severe lactose intolerance in infancy was found [138]. In the second half of twentieth century, it was found that the lactose intolerance was genetically-determined [139]. Nowadays, many options were used to prevent the abdominal and gastrointestinal symptoms of lactose intolerance [140, 141].

In addition, some health professionals not advising the consumption of milk because it could cause an inflammatory process. However, there was no evidence showed the association. Recently, several publications have shown that milk and dairy production consumption were not related to the inflammatory response [142–144]. A systematic review of 15 latest RCTs evaluated the scientific evidence of the effects of milk on inflammatory bio-markers, and found that consumption of milk did not show a pro-inflammatory effect in healthy subjects or individuals with metabolic abnormalities (who were obese, overweight or who had T2DM or metabolic syndrome) and even had a significant anti-inflammatory effect in both healthy and metabolically abnormal subjects [142].

### Strengths and limitations

The umbrella review systematically summarized the current evidence for milk intake and a range of health-related outcomes for humanity. The AMSTAR 2 and GRADE were used to assess the quality of methods and the evidence for each included meta-analyses. However, several possible limitations should be considered. The article with pooled analysis were included. Those without meta-analyses were omitted, which would have impacts on the outcomes. Besides, we are unable to analyze the associations of different types of milk (whole/high-fat/low-fat/skimmed) with individual outcomes, because most of the articles did not distinguish the different types of milk. In addition, most of the outcomes came from observational study, which may limit the association effects for each outcome due to heterogeneity and bias across studies [145]. Since this umbrella review aim to investigate the association of milk consumption and health outcomes, the physiological outcomes were omitted. In addition, some studies showed that there was a dose dependent effect, while we were unable to conduct the dose–response analysis, more work should be done to elucidate the dosage and effects of milk consumption on human health.

## Conclusions

Milk consumption has been investigated for association with a diverse range of health outcome in a large amount of meta-analyses. In this umbrella review, milk consumption does more good than harm for human health. Our results support milk consumption as part of a healthy diet. More well-designed RCTs are warranted in the future.

## Supplementary Information

**Additional file 1.** Table S1: Full versions of total summary data for the meta-analyses of association between milk consumption and health outcomes.

**Additional file 2.** Table S2: The detailed results of AMSTAR 2 of each meta-analysis.

## Data Availability

Not applicable.

## Abbreviations

MPL: Milk polar lipids
ALA: α-Linolenic acid
CLA: Conjugated linoleic acids
OR: Odds ratio
RR: Relative risk
HR: Hazard ratio
MD: Mean difference
SMD: Standardized mean differences
RCTs: Randomized controlled trials
CI: Confidence interval
AMSTAR: A measurement tool to assess systematic reviews
GRADE: Grading of Recommendation Assessment, Development and Evaluation
CVD: Cardiovascular disease
CHD: Coronary heart disease
T2DM: Type 2 diabetes mellitus
AD: Alzheimer's disease
PD: Parkinson’s disease
BP: Blood pressure
TC: Total cholesterol
SFAs: Saturated fatty acids
LDL-C: Low density lipoprotein cholesterol
HDL-C: High density lipoprotein cholesterol
Apo: Apolipoprotein
MA: Meta-analysis
FFAs: Free fatty acids
NHL: Non-Hodgkin’s lymphoma
HCC: Hepatocellular carcinoma
CRC: Colorectal cancer
DLBCL: Diffuse large B-cell lymphoma
FL: Follicular lymphoma
SLL/CLL: Small lymphocytic lymphoma/chronic lymphocytic leukemia
ESCC: Esophageal squamous cell carcinoma
NA: Not available
PC: Prostate cancer
FDA: Fe-deficiency anaemia
HTG: Hypertriacylglycerolaemia
T1DM: Type 1 diabetes mellitus
